# Supplementary material for: Connexin Hemichannel Activation by S-Nitrosoglutathione Synergizes Strongly with Photodynamic Therapy Potentiating Anti-Tumor Bystander Killing
Source: Cancers (Basel). 2021 Oct 10;13(20):5062. doi: 10.3390/cancers13205062 (PMC8533914; doi:10.3390/cancers13205062)
Supplement: Supplementary file 1 [file cancers-13-05062-s001.zip › cancers-1419321-supp-final-updated.pdf]

Supplementary Materials

Connexin Hemichannel Activation by S-Nitrosoglutathione Synergizes Strongly with Photodynamic Therapy Potentiating Anti-Tumor Bystander Killing

Chiara Nardin, Chiara Peres, Sabrina Putti, Tiziana Orsini, Claudia Colussi, Flavia Mazzarda, Marcello Raspa, Ferdinando Scavizzi, Anna Maria Salvatore, Francesco Chiani, Abraham Tettey-Matey, Yuanyuan Kuang, Guang Yang, Mauricio A. Retamal and Fabio Mammano

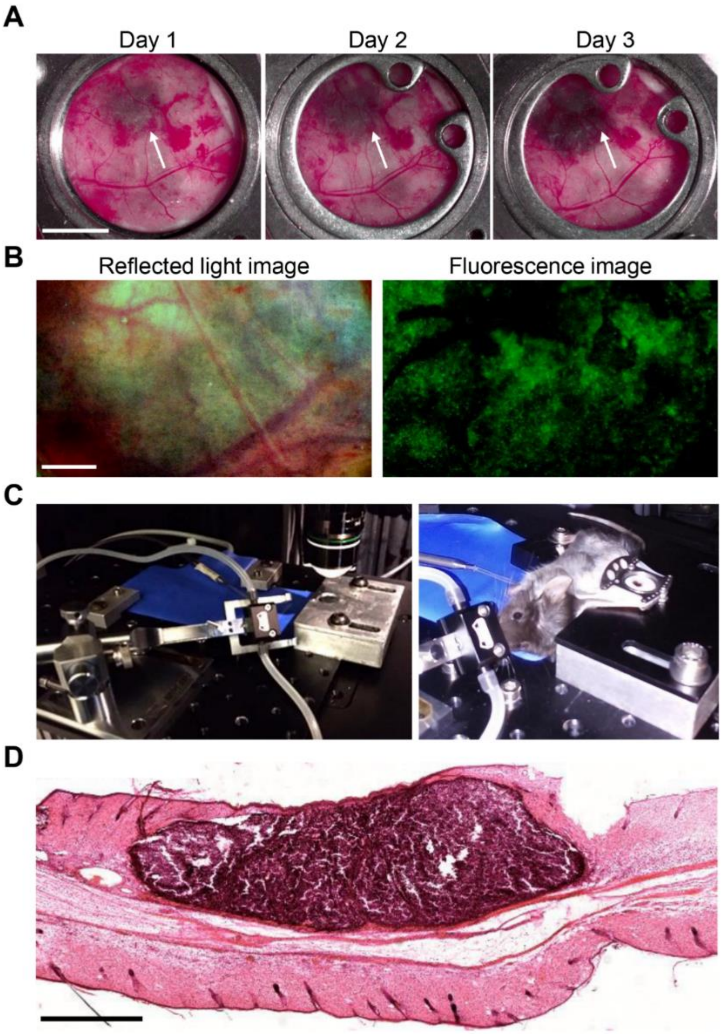

Figure S1. Development of fluorescent indicator-expressing tumors for intravital imaging in the dorsal skinfold chamber (DSC). (**A**) Reflected light images of pigmented B16-F10 melanoma cells growing in the DSC at shown time points after seeding (day 0). The peritumoral region marked with the white arrow is progressively invaded by melanoma cells; scale bar: 0.5 cm. (**B**) Detail of fluorescent melanoma-bearing tissue. Left: reflected light image. Right: fluorescence image of GCaMP6s-expressing melanoma cells; scale bar: 1 mm. (**C**) Multiphoton microscope stage adapted for intravital microscopy. Left: stage with mouse heating pad, gaseous anesthesia supply and DSC holder. Right: detail of a mouse bearing the DSC fixed to the microscope stage. (**D**) Representative transverse section of tissue sample bearing a B16-F10 tumor grown in the DSC, harvested 5 days after cell seeding and stained with hematoxylin and eosin; scale bar: 1 mm.

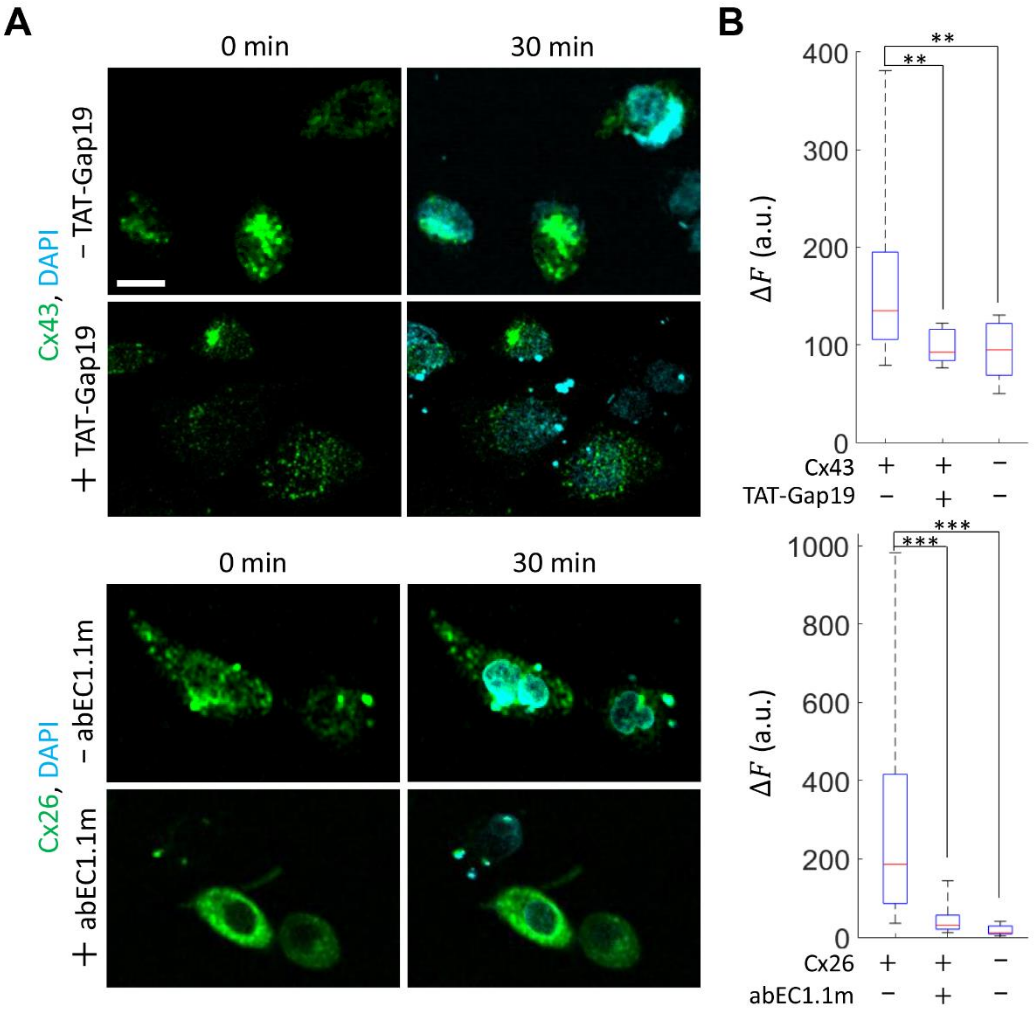

Figure S2. Blockers of connexin 43 (Cx43) and connexin 26 (Cx26) hemichannels (HCs) reduce 4′,6-Diamidine-2′-phenylindole dihydrochloride (DAPI) uptake in HeLa DH transfectants. HeLa DH cells expressing Cx43 (top) or Cx26 (bottom) fused to the fluorescent protein Venus (green) were incubated with DAPI dissolved in calcium-free extracellular medium (CFEM) to promote HCs opening. (**A**) Representative images acquired before (t = 0 min) and after 30 min of incubation with DAPI (5 μM) in the presence (+) or in the absence (−) of TAT-Gap19 (150 μM, top) or abEC1.1m (1 μM, bottom), which block Cx43 and Cx26 HCs, respectively; scale bar: 20 μm. (**B**) Box plots showing the distributions of $\Delta F = F(30\text{ min}) - F(0\text{ min})$ fluorescence intensity of DAPI measured in $n \geq 15$ nuclei in the presence (+) or in the absence (−) of TAT-Gap19 (top) or abEC1.1m (bottom). In both graphs, data obtained in non-transfected HeLa DH cells exposed to CFEM are shown in the box plot on the right. Red horizontal bars represent the median; bottom and top edges of the blue boxes indicate the 25th and 75th percentiles, respectively; black tails of the boxes mark the most extreme data points in the distribution. a.u., arbitrary units; **, $p < 0.01$; ***, $p < 0.001$, the Mann-Whitney U test.

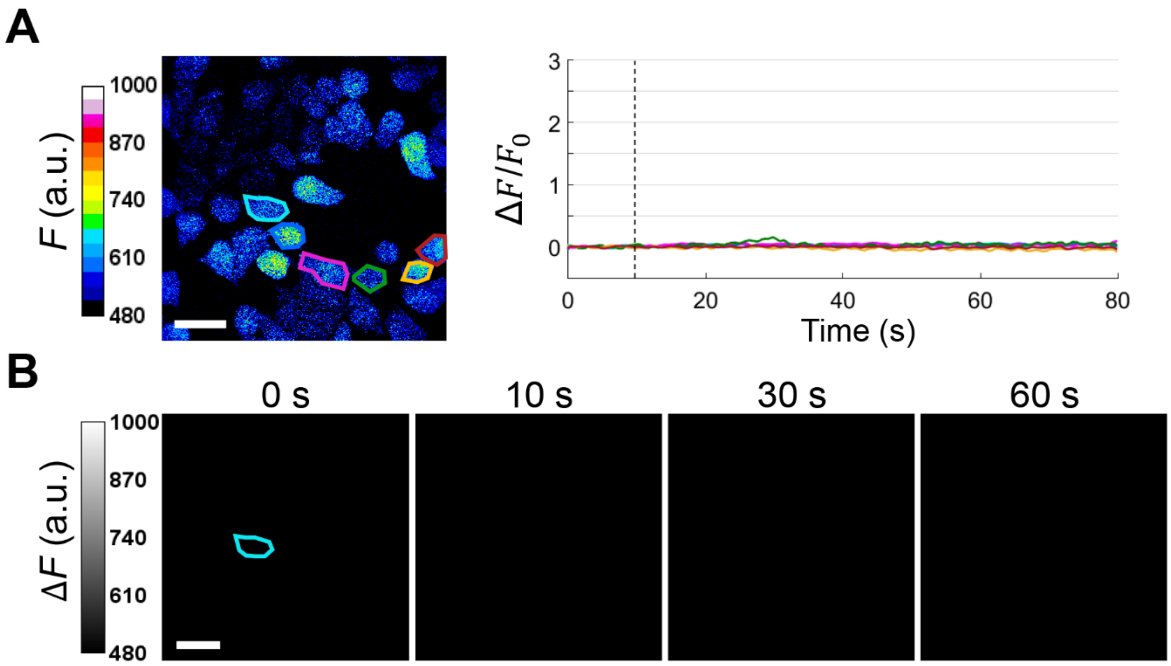

Figure S3. Single-cell continuous irradiation in a whole-cell ATP biosensor (ATP-WCB) culture. (**A**) False-color fluorescence (*F*) image of an ATP-WCBs culture loaded with Fluo-8H. The cell marked with the cyan region of interest (ROI) was exposed to continuous irradiation for 70 s by the focused photoactivation laser (671 nm, ~5 × 10^6 mW/cm^2). The graph on the right shows single-cell $\Delta F/F_0$ traces ($\Delta F = F - F_0$, where F_0 = pre-stimulus value) generated as pixel signal average within color-matched ROIs shown on the left. The vertical dashed line marks the onset of laser irradiation (t = 10 s). (**B**) Fluorescence intensity variations (ΔF) of cells shown in (**A**) at different time points after the onset of focal irradiation of the cell marked with the cyan ROI. Results are representative of n = 3 independent trials. a.u., arbitrary units; scale bars: 20 μm.

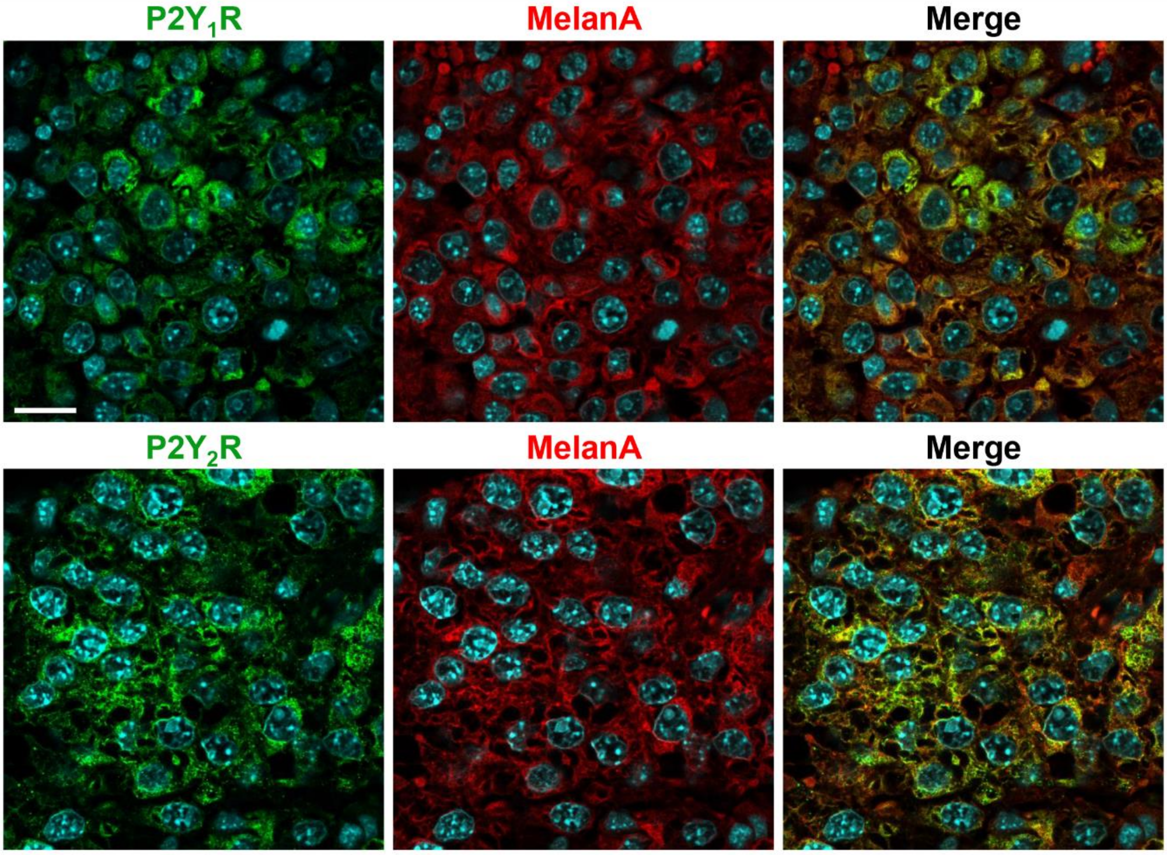

Figure S4. P2Y1 and P2Y2 receptors (Rs) are expressed in B16-F10-derived tumors. Confocal fluorescence images obtained by immunostaining with antibodies selective for P2Y1R (top left, green), P2Y2R (bottom left, green) and MelanA (middle, red) in representative sections of melanomas grown in dorsal skinfold chambers; nuclei were stained with 4′,6-Diamidine-2′-phenylindole dihydrochloride (DAPI, cyan); scale bar: 20 μm.

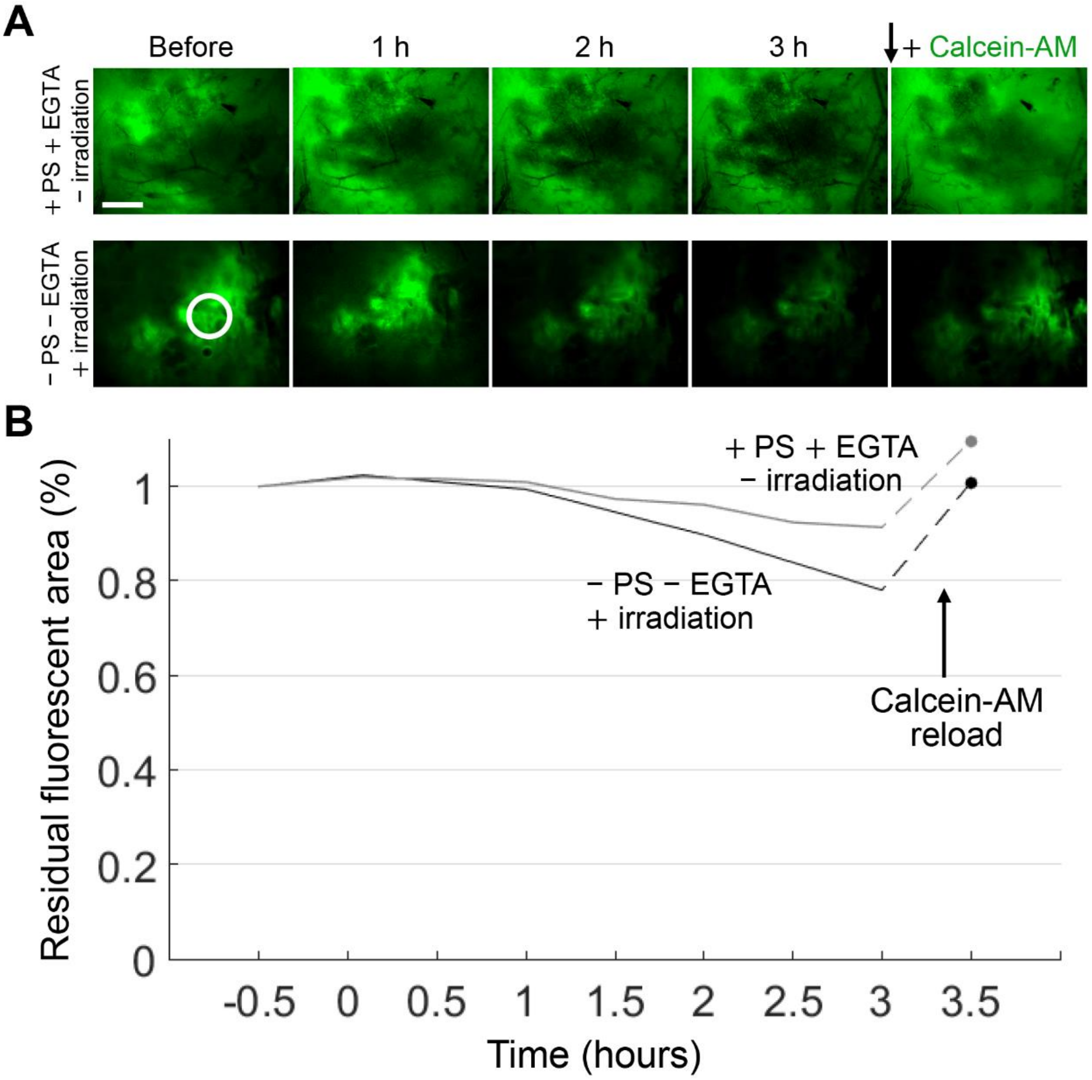

Figure S5. Calcein-AM tumor cell viability assays in control conditions. B16-F10 melanoma cells grown inside the dorsal skinfold chamber were loaded with green fluorescent viability dye calcein-AM (see Figure 4B,C). (**A**) Fluorescence images acquired at different time points after the following treatments: microinjection of photosensitizer (PS) and ethylene glycol-bis(β-aminoethyl ether)-N,N,N′,N′-tetraacetic acid (EGTA, 5 mM) dissolved in Ca^{2+}-free extracellular medium, without irradiation (+PS +EGTA −irradiation, top); irradiation (within the white circle) in the absence of both PS and EGTA (−PS −EGTA +irradiation, bottom); the black down arrow marks the time point of calcein-AM reloading in the tumors (3 h 30 min); scale bar: 1 mm. (**B**) Time-dependent variation of the area with persistent calcein-AM fluorescence signal after treatment in +PS +EGTA −irradiation (grey trace) or −PS −EGTA +irradiation (black trace) conditions. Solid lines represent continuous time-lapse image acquisition, dashed lines represent the final value obtained after calcein-AM reloading in the tumors.

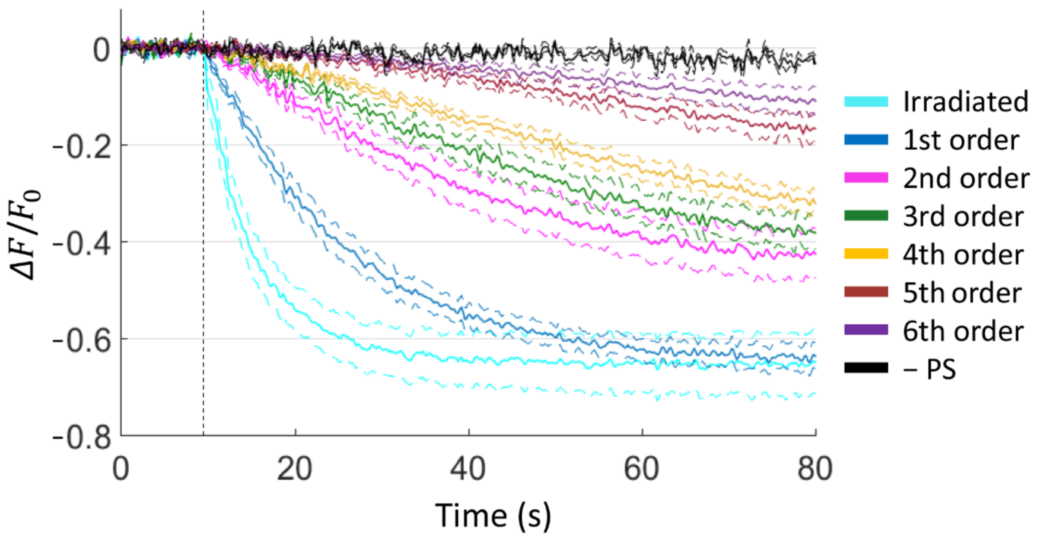

Figure S6. Kinetics of caspase-3 (Cas-3) activation in B16-F10 cells exposed to focal photodynamic therapy in vitro. Shown are mean (solid lines) ± standard error of the mean (s.e.m., dashed lines) Cas-3 indicator $\Delta F/F_0$ signals from irradiated and bystander cells (from the 1st to the 6th order); pooled data from $n = 6$ experiments performed in normal extracellular medium. The black trace is the signal (mean ± s.e.m.) obtained from irradiated cells in the absence of photosensitizer (−PS, negative control). The vertical dashed line marks the onset of laser irradiation ($t = 10$ s).

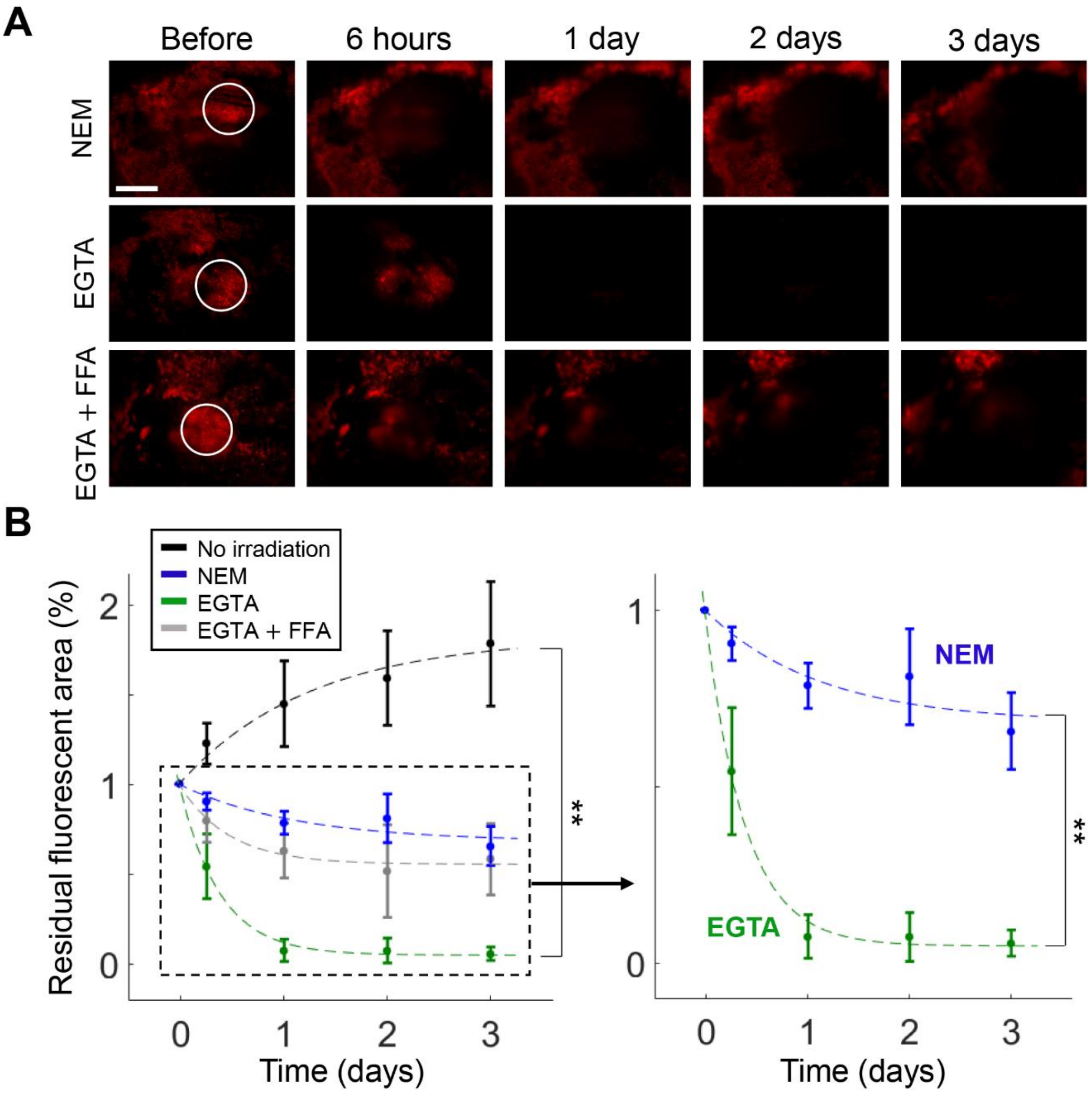

Figure S7. Activation of connexin hemichannels by intratumor injection of ethylene glycol-bis(β-aminoethyl ether)-N,N,N′,N′-tetraacetic acid (EGTA) increases bystander cell death following spatially confined photodynamic therapy (scPDT). (**A**) Representative results of time-lapse experiments. Fluorescence images were acquired before and after scPDT (within white circles) at shown time points (see Materials and Methods); scale bar: 1 mm. (**B**) Left: time-dependent variation of the area with persistent mCherry fluorescence signal after scPDT in: normal extracellular medium (NEM, blue); Ca^{2+}-free extracellular medium (CFEM) supplemented with 5 mM EGTA (EGTA, green); CFEM supplemented with 5 mM EGTA plus 100 μM of flufenamic acid (EGTA+FFA, gray). No irradiation (black) refers to data obtained in the presence of photosensitizer but in the absence of irradiation. Shown are mean values ± standard error of the mean of $n \geq 3$ independent replicates for all tested conditions. Interpolating functions (dashed lines) were computed by data fitting with the model: $f(t) = 1 - a + ae^{-b(t+c)}$ (for parameter values see Table S3). **, $p < 0.01$, ANOVA (for post hoc pairwise comparisons see Table S4). Right: detail of the results obtained in NEM (blue) and CFEM supplemented with 5 mM EGTA (green); **, $p < 0.01$, two-tailed t-test.

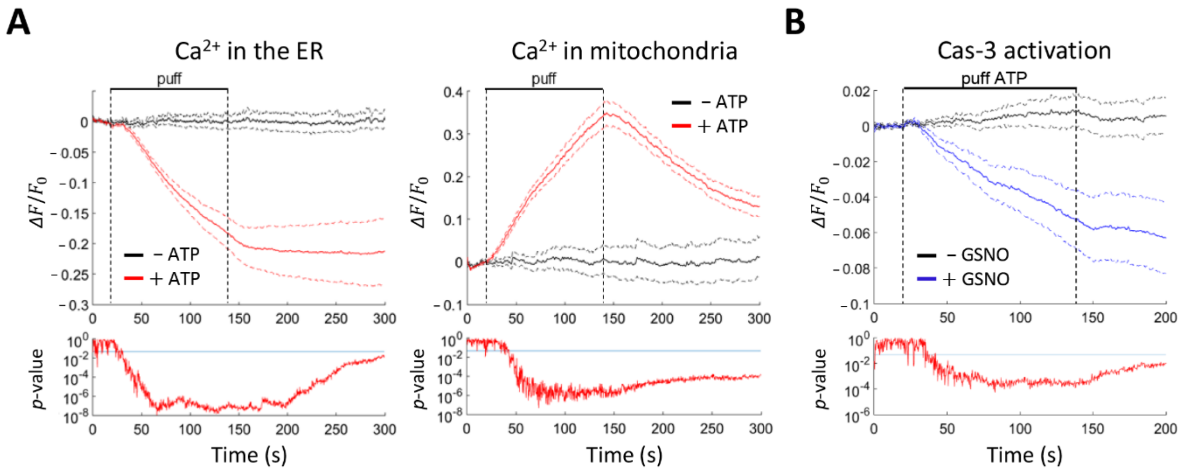

Figure S8. Responses of B16-F10 cells to exogenous adenosine triphosphate (ATP, 100 nM) applied by pressure through a glass microcapillary in calcium (Ca^{2+})-free extracellular medium. (**A**) Application of ATP (red traces) promotes Ca^{2+} release from the endoplasmic reticulum [ER, left, average $\Delta F/F_0$ signal (solid lines) ± standard error of the mean (s.e.m., dashed lines) obtained from $n \geq 5$ G-CEPIA1er-expressing cells] and mitochondrial Ca^{2+} uptake [right, average $\Delta F/F_0$ signal (solid lines) ± s.e.m. (dashed lines) obtained from $n \geq 10$ CEPIAmt2-expressing cells]. The black traces are the results of negative control experiments in which ATP was omitted from the pressure-applied medium. (**B**) ATP triggers caspase-3 (Cas-3) activation only in cell cultures that had been pre-incubated for 50 min with S-Nitrosoglutathione [GSNO, 100 nM, blue traces, average $\Delta F/F_0$ signal (solid lines) ± s.e.m. (dashed lines) obtained from $n \geq 7$ cells expressing the fluorescent indicator for Cas-3 activity]. Black traces are the result of ATP application experiments in the absence of GSNO. Vertical dashed lines mark the onset and the end of stimulus delivery (from $t = 20$ s to $t = 145$ s). At the bottom of each graph, point-by-point p-values (two-sample t-test) between the curves obtained in the two conditions are shown on a log scale; horizontal blue lines mark the level of statistical significance ($p = 0.05$).

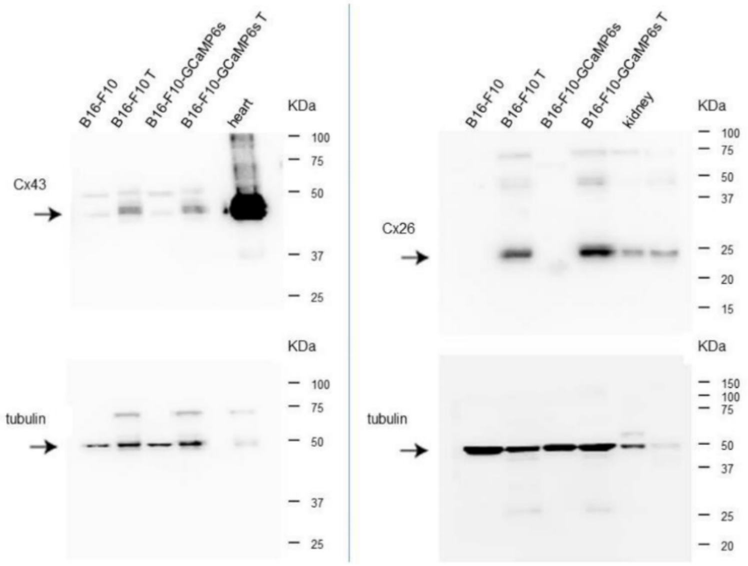

B

Samples	Cx43 O.D.	tubulin O.D.	Relative O.D. Cx43/tubulin
B16-F10	5042155	8353887	0.60
B16-F10 T	22053221	13404533	1.65
B16-F10-GCaMP6s	4088919	9892826	0.41
B16-F10-GCaMP6s T	21756707	14785829	1.47

Samples	Cx26 O.D.	tubulin O.D.	Relative O.D. Cx26/tubulin
B16-F10	0	3910365	0
B16-F10 T	4117620	9950086	0.41
B16-F10-GCaMP6s	0	5783863	0
B16-F10-GCaMP6s T	8645995	16264796	0.53

C

Samples	Cx43/tubulin		Cx26/tubulin	
	Mean relative O.D.	s.e.m.	Mean relative O.D.	s.e.m.
B16-F10	0.43	0.11	0	0
B16-F10 T	1.603	0.054	0.425	0.012
B16-F10-GCaMP6s	0.53	0.16	0	0
B16-F10-GCaMP6s T	1.69	0.21	0.483	0.038

Figure S9. Original images and densitometry readings for blots of Figure 2E. (**A**) Full representative western blot images for Connexin 43 (Cx43, left) and Connexin 26 (Cx26, right) expression in tumors (T) derived from B16-F10 or B16-F10-GCaMP6s cells and grown in dorsal skinfold chambers (denoted as B16-F10 T and B16-F10-GCaMP6s T, respectively) compared with B16-F10 or B16-F10-GCaMP6s cells grown in culture dishes. (**B**) Values of measured optical density (O.D.) for the bands shown in (A). (**C**) Mean values for the relative O.D. ± standard error of the mean (s.e.m.) computed from $n = 4$ independent experiments for both Cx43 and Cx26 expression (see Figure 2E).

Table S1. Statistical analysis for in vivo calcium (Ca^{2+}) waves induced by focal photodynamic therapy (fPDT) in the presence of connexin hemichannel (HC) blockers (Figure 2A).

HC blocker	1st order	2nd order	3rd order	4th order	5th order	6th order
NEM	0.11	**0.018**	**0.012**	**4.9 × 10^−3^**	0.52	0.56
CBX	0.76	**0.049**	0.19	**1.9 × 10^−3^**	0.13	0.93
FFA	0.44	**2.1×10^{-4}**	**2.6×10^{-7}**	**3.0×10^{-7}**	**7.2×10^{-5}**	**8.8×10^{-3}**
TAT-Gap19	0.52	**3.2×10^{-3}**	**7.7×10^{-14}**	**$<10^{-15}$**	**1.0×10^{-11}**	**2.1×10^{-6}**
abEC1.1m	0.066	**1.1×10^{-3}**	**6.5×10^{-3}**	**7.4×10^{-6}**	**2.3×10^{-4}**	0.19

p-values obtained by pairwise statistical comparisons with post hoc Dunn-Sidak correction after Kruskal-Wallis test on fPDT-triggered bystander Ca^{2+} wave amplitudes. Reported values are referred to results obtained in the presence of ethylene glycol-bis(β-aminoethyl ether)-N,N,N′,N′-tetraacetic acid (EGTA conditions, see Figure 2A). NEM, normal extracellular medium = no HC blocker; CBX, carbenoxolone; FFA, flufenamic acid. Values in bold are < 0.05.

Table S2. Statistical analysis for in vivo 4′,6-Diamidine-2′-phenylindole dihydrochloride (DAPI) uptake experiments of Figure 2D.

HC blocker	EGTA	NEM	FFA	TAT-Gap19
NEM	**1.9×10^{-4}**	-	-	-
FFA	**5.8×10^{-6}**	1.0	-	-
TAT-Gap19	**0.002**	1.0	1.0	-
abEC1.1m	**0.042**	0.80	0.48	0.98

p-values obtained by pairwise statistical comparisons with post hoc Dunn-Sidak correction after Kruskal-Wallis test for in vivo DAPI uptake results obtained in the presence of connexin hemichannel (HC) blockers (see Figure 2D). NEM, normal extracellular medium = no HC blocker; CBX, carbenoxolone; FFA, flufenamic acid. Values in bold are < 0.05.

Table S3. Fitting function and parameter values for data fitting in Figure 4B and S7B.

$\mathbf{f}(t) = 1 - a + ae^{-b(t+c)}$	**Calcein-AM [*t*] = hours**		**mCherry [*t*] = days**			
	**NEM**	**EGTA**	**NEM**	**EGTA**	**EGTA + FFA**	**No irradiation**
a	1.078	1.034	0.3196	0.9525	0.4462	−0.8178
b	0.1687	0.7737	0.8875	2.578	2.1	0.7871
c	0.5	0.5	0.0208	0.0208	0.0208	0.0208
R-squared	0.982	0.9972	0.8598	0.9962	0.9775	0.9736

Table S4. Statistical analysis for spatially confined photodynamic therapy (scPDT) experiments of Figure S7B.

scPDT condition	6 hours	1 day	2 days	3 days
NEM	0.54	0.19	0.21	0.086
EGTA	**0.016**	**2.5×10^{-3}**	**4.7×10^{-3}**	**6.5×10^{-3}**
EGTA + FFA	0.19	0.071	**0.043**	0.062

p-values obtained by pairwise statistical comparisons with post hoc Bonferroni correction after ANOVA test on results of scPDT experiments performed in normal extracellular medium (NEM), ethylene glycol-bis(β-aminoethyl ether)-N,N,N′,N′-tetraacetic acid (EGTA) conditions and EGTA plus flufenamic acid (EGTA + FFA) conditions compared to no irradiation conditions (see Figure S7B). Values in bold are < 0.05.

Table S5. Fitting function and parameter values for data fitting in Figure 6B.

$f(t) = 1 - a + ae^{-b(t+c)}$	−GSNO	+GSNO	+GSNO +TAT-Gap19	+GSNO +abEC1.1m
a	0.2246	0.8145	0.4465	0.08843
b	13.46	4.211	14.17	13.79
c	0.0417	0.0417	0.0417	0.0417
R-squared	0.51	0.9992	0.9852	0.1225

Table S6. Statistical analysis for full photodynamic therapy (PDT) experiments of Figure 6D.

Full PDT Condition	−GSNO	+GSNO	+TAT-Gap19	+abEC1.1m
−GSNO	-	-	-	-
+GSNO	**0.044**	-	-	-
+TAT-Gap19	0.80	0.59	-	-
+abEC1.1m	0.99	**0.018**	0.59	-

p-values obtained by pairwise statistical comparisons with post hoc Dunn-Sidak correction after Kruskal-Wallis test between tumor volumes from full PDT experiments performed in the following conditions: absence of S-Nitrosoglutathione (−GSNO); presence of GSNO (+GSNO); presence of GSNO plus TAT-Gap19 (+TAT-Gap19) or abEC1.1m (+abEC1.1m) (see Figure 6D). Values in bold are < 0.05.

© 2021 by the authors. Licensee MDPI, Basel, Switzerland. This article is an open access article distributed under the terms and conditions of the Creative Commons Attribution (CC BY) license (http://creativecommons.org/licenses/by/4.0/).
